# Supplementary material for: Effectiveness of short-term isothermic-heat acclimation (4 days) on physical performance in moderately trained males
Source: PLoS One. 2022 Nov 17;17(11):e0270093. doi: 10.1371/journal.pone.0270093 (PMC9671432; doi:10.1371/journal.pone.0270093)
Supplement: Attachment — Submitted filename: PLoS One.pdf [file pone.0270093.s002.pdf]

General Comments

The authors completed a 4 day, 90 min isothermic HA study, with heat stress tests completed before, and following, the HA period. I have found the study design to be vague and challenging to understand and have considerable reservations regarding some of the statistical methods. In short, I believe that the authors should consider expanding the methodological section, rechecking the statistical outcomes and considerably tempering language used – the outcomes of this study are weakened by the lack of a thermoneutral/temperate control group, yet this is not discussed. There are also quite a few issues with spelling and grammar throughout the paper.

Introduction

I believe that the introduction could be improved by expanding upon the methodology and procedures behind controlled hyperthermia. Line 54-56 does not provide a reader with enough insight into what the methodology actually is, and how it is achieved, and most importantly, how it differs from other HA protocols. Further, it would be of benefit to discuss the often cited threshold temperature of 38.5C (something that is relevant for the present paper as well), and how dehydration increases thermal strain, especially as this is used in your study.

Please include more information around the physiological processes that drive the HA adaption as well.

Line 53 – remove the word 'typical' as I would argue that isothermic is simply one methods for HA

Line 61 – provide reference

Line 64 – the term 'limited evidence of efficacy' suggests that the evidence doesn't support 4 day exposure. I don't believe your trying to say this? Aren't your trying to say that there just isn't much research on 4d HA, at all? Rewrite

Methods

The methodology section is currently insufficient and is lacking in necessary detail. Please see specific comments below.

Experimental design paragraph – I found this a challenge to understand the design of the study. For example, what was the length of time between the end of HA and the HST3? The schematic is too vague to be of assistance here. Please consider revising the figure and have a look at similar HA papers for an idea of different designs schematics. Indicate when measures were collected during a HST and HA session too.

Line 85 – what was the average temp and humidity range across the summer period when data collection occurred?

Line 87 – insert the word exercise after strenuous

Line 87 - Reword the sentence 'further caffeine and alcohol….' It does not make sense at the moment.

Line 92 – specific the cycle ergometer used

Line 92-98 – how was VO2peak and PPO determined? Average over the last stage? Last measurement prior to failure? Rolling 15s average? Etc

Line 101 – was the climate chamber temperature/humidity recorded with another device as well? What was the SD of these measures over the HA period? Similarly for the HSTs?

Line 102-103 – more information is required around he cycling protocol for isothermic HA. Participants were told to cycle at a 'comfortable pace' but also to reach 38.5C as quickly as possible? What was the resistance? Was there a max time limit that a participant had to reach 38.5C in? as the faster they reached that point, the longer duration in the adaption zone, so ensuring this was consistent is important. The time to 38.5C on day 1 was SD of ~7 min calc, or ~7% variance. With such a short duration protocol of 4 days, is it possible that this variation may have altered the outcome, through masking physiological responses between participants?

Perceptual measures are discussed in the results, but aren't mentioned in the methods at all? What scales were used, and when were they collected?

Line 106 – comma needed between 2011 and 2014

Line 109 – change semi colon to full stop

Line 108 – but when was the urine collected? Immediately prior to exercise? Upon waking? After exercise? Etc

When was body mass collected? Was it a nude body mass? Post exercise, did the participants towel dry? What did participants wear during the trials, and was it consistent?

Line 114-118 – How were these measures analysed though? ELISA? Provide the devices, techniques and CV% (if relevant)

When were Hb and Hct collected? When was venepuncture completed?

Line 125 – what model of bike was it though?

Line 130 – consider changing the term cardiac frequency to heart rate. This is considerably more commonly used in exercise physiology research and will be easier for a reader to understand.

Line135 – thermistors doesn't need to be capitalised and please specific the number of sites used (4? 8? etc)

Results

Line 163 – 165 – Certainly time to reach 38.5C increased but since power output wasn't fixed, isnt it possible that participants simply cycled slower? Although there wasn't any difference in total work completed, the massive variation makes it difficult to tell.

Table 1 – add in duration above 38.5C in minutes to the table

Only n = 10 completed the HA? This should be stated from the start then, and needs to be changed throughout the document and abstract.

I am confused regarding the terminology being used to explain the main effects throughout the results section. For example, the urine measures ANOVA would be 'day + time + day x time', with follow-up post hoc tests when a sig main effect is found. The term main effect, should be sued for all

primary factors, so in the previous example, that would be ‘day’ and ‘time’. Please change this throughout the paper.

However, it appears that post-hoc test have been run when no interaction effect was found, yet presented as such? E.g., Line 179-178 states that $colour_u$ had a sig main effect for time (and therefore, not for day or interaction of day x time). Yet it is then further investigated for day 1 pre-post and day 4 pre-post which is no necessary (as day was not significant). This error is continuous throughout the article; perhaps I have not fully understood what was being compared here? Please explain the reason for this.

Another example is 198-200 – I assume that the ‘no main effect for T_{re}’ is referring to HST2vs3? Therefore, please ensure that it is understood that the decrease in T_{re} at 70min is independent of the HA, i.e., between HSTs. This should be more accurately indicated on Figure 4, as it currently appears as if there was an interaction effect and a resulting posthoc test that shows a sig difference at 70mins between the HST, which is not the case.

Line200-201 – How was Tre reduced at rest and 90min, if the previous analysis only found a difference at 70mins?

Line 208-2013 – same issues as previously mentioned. I’m confused what analysis is occurring here. Based on what is written in the test, there was only a main effect of time (unsurprising since it will have increased due to exercise, regardless of which HST). However, the way the figure is present and written in text it appears that the post hoc test was conducted as if there was an interaction effect – which I dont believe there was? At least that isn’t stated.

Line 213 – provide this data in table format too

Perceputal paragraph - this is the first-time perpetual measures have been mentioned. They should have been discussed in the methods section.

Discussion and Conclusion

Line 238 – but it wasn’t on 12 males, its was on….10? maybe 11?

Line 238 – no, I don’t believe that post intervention that we did see decreases in those measures. We saw differences across time, but as there was no sig interaction effect (at least, its not stated?) then the post hoc tests can’t be run. I required more information to understand this, and am interested to hear the outcome.

Line 239-241 – this language should be strongly tempered. There was no control for this study, and as such, it is impossible to say if any improvised pre-post HA were due to HA, or were simply due to the training effect. In other words, maybe these participants may have got a similar effect if they had trained in 20C – we don’t know, and as such, cannot state what is written there.

Paragraph starting at Line 243 – this entire section appears disjointed and is not suitable in its present format. Please consider rewriting this section to be more focused on the outcomes of the present study, and the context in which they sit.

Line 287 – but again, if there was no interaction, than the posthoc should not have been run for this.

Line 296 - Add in a full stop.

Line 301 – remove capitalisation

Line 308 – 309 – but depending on when bloods were taken, this may alter PV due to hydration

Linr310 – is the training status of athletes affects the magnitude of PV expansion, than why did this not occur in the present study (with lower fitness level participants)?

Line 311 – remove comma, add ‘and’

Line 313 – change word ‘beliefs’

Line 315 – shouldn’t have reference number, have author surname instead. Same on Line 316

The remainder of the discussion is heavily focused on blood parameters. I believe that this should be rewritten in a more succinct manner and focused primarily on the outcomes of the present study. I would have also imaged a limitations section is necessary here too?
