# Supplementary material for: Effectiveness of short-term isothermic-heat acclimation (4 days) on physical performance in moderately trained males
Source: PLoS One. 2022 Nov 17;17(11):e0270093. doi: 10.1371/journal.pone.0270093 (PMC9671432; doi:10.1371/journal.pone.0270093)
Supplement: Attachment — Submitted filename: Plos One - Jefferson F.C. Rodrigues Junior.docx [file pone.0270093.s001.docx]

Dear authors, congratulations on your study, which was carefully conducted and present adequate controls. The following comments are aimed to clarify and improve the manuscript, including information that could be added in the revised version.

How did you test 12 individuals? Did the authors made a priori sample size calculation (if so, please add this calculation)? Alternatively, was this sample size obtained as a convenience, or was it based on the number of participants in similar studies?

Were the experimental procedures carried out using each participant's own bicycle or did the researchers provide one same bicycle for all of them?

Since generally trained people exercise outdoors, did you familiarized the subjects with exercising indoors? If so, please inform the duration, speed/intensity and environmental conditions during the familiarization sessions. If not, please justify why this familiarzation was not required.

Because many people are not familiar with the perception scales, were the subjects previously familiarized with their use?

Please include a paragraph with the limitations of your study.

The results indicate that a short-term acclimatization period induced several adaptations, either physiological or perceptual adaptations. However, this reviewer suggests that future experiments should include a performance-oriented test (in addition to heat-stress tests), such as the countermovement jump. For example, the countermovement jump which could be used could be performed before and after heat stress testing session to evaluate whether there was a decrease in lower limb power induced by the test and whether acclimation will influence this decrease.
