# Supplementary material for: Effectiveness of short-term isothermic-heat acclimation (4 days) on physical performance in moderately trained males
Source: PLoS One. 2022 Nov 17;17(11):e0270093. doi: 10.1371/journal.pone.0270093 (PMC9671432; doi:10.1371/journal.pone.0270093)
Supplement: Attachment — Submitted filename: JSHAWPLOSONERESPONSE1.docx [file pone.0270093.s003.docx]

**Reviewer #1:**

**Reviewers’ Comment #1:**

“Dear authors, congratulations on your study, which was carefully conducted and present adequate controls. The study presented robust methodological data well designed with a design. The questions were answered, in view of that…”

**Authors’ Response:**

*We thank you for your encouragement and appreciate your feedback to make this manuscript the best it can possibly be.*

**Reviewer #2:**

**Reviewer’s comment #1:**

“Line 43: why is an exact p-value provided in line 42, but P<0.05 used here? Same with the lines further on in the abstract”

**Authors’ response:**

*We thank you for your comment. We intended to show that all the measurements were significant <0.05 as opposed to writing out 3 separate p values. However, we appreciate your comment and perspective, and this has been corrected. Please see lines 43&44.*

**Reviewer’s comment #2:**

“Line 161: I believe the word should be ‘inverted’ not ‘invested’?”

**Authors’ response:**

*We thank you for your thoroughness and you would be correct. This has been rectified on line 162.*

**Reviewer’s comment #3:**

“I previously suggested that the use of ‘cardiac frequency’ in rare and potentially confusing to readers. This was agreed in the response to my comments, but this change has not been undertaken throughout the document?”

**Authors’ response:**

*We thank you for your critique with this issue and this has been rectified throughout the document.*

**Reviewer’s comment #4:**

“Line 173: Apologies if I wasn’t clear in my previous comment, but I meant please calculate and provide the intraassay CV (and inter- if you used multiple plates for the same variable) of each bloods variables from the ELISA plates”.

**Author’ response:**

*We appreciate your comment and have opted to keep the contents of the table as it is as CoV is a construct of mean and standard deviation. A keen reader can always calculate it if they wished using (SD/Mean)*100. We have included the manufacturer CoV directly.*

**Reviewer’s comment #5:**

“Line 199: I am pretty sure this should say ‘6-20’ scale, not 1-20?”

**Authors’ response:**

*We thank you for your thoroughness and have rectified this on line 203.*

**Reviewer’s comment #6**

“Data analysis section: how was missing data handled?”

**Authors’ Response:**

*We thank you for your comment. Where data was missing, this is indicated by “n=” where appropriate. With regards the ANOVA analysis and the HST section, these were originally ran as paired. Consequently, this meant that certain participants would be excluded if they did not complete the full 90-minutes. This was the only period where data would have been affected. To avoid participant exclusion where a participant dropped out of the HST before 90-min, their final measurement was duplicated into missing time points to include all participants. This resulted in greater degrees of freedom and only slightly altered the p-values. No outcome measures became significant where not statistically significant previously (and vice-versa). Therefore, this did not affect the overall interpretation results. We have updated the statistics to reflect an n=12* *throughout the HST section. The data analysis section has been updated to reflect these changes.*

**Reviewer’s comment #7:**

“Based on what is written in the HST section of the results, Tre and HR saw no main effects for day or an interaction, just time. This makes sense. But then the subsequent sentences (Line 248-250 and 257-259) discuss that rest and end-exercise Tre and HR were reduced…and seems this means ‘reduced compared to Day 1’. Which is not what is described in the analysis, as it has already been indicated that there was no interaction effect? So really it should only be demonstrating differences in time, e.g., rest vs endpoint (which will obviously be sig. different). So, I am a little confused on what these comparison are referring to?

This is highly important as it is a central premise of this paper. For example, Line 319-20 and 338-340 in the discussion.”

**Authors’ response:**

*We appreciate your comment. We have kept the descriptive statistics in the results in order for the discussion to be informed by the data.*

**Reviewer’s comment #8:**

“Line 247 – states Fig 5 upper, think it should be Fig 4”

**Authors’ response:**

*We thank you for your comment and this has been rectified on line 274.*

**Reviewer’s comment #9:**

“As stated above, I do not fully understand about the statements at the start of the Tre and HR sections, regarding resting measures. For example, the reductions in resting Tre mentioned in Line 324 appear to refer to significant reduction in this measure between pre- and post-HA (i.e., effect of the HA intervention). But in the results section this is discussed as if it was a t-test of rest and 90min pre-HA vs post-HA, yet the preceding sentences specify that there was only a main effect for time (and not day x time).

The large variance of both of these resting changes do not exactly support the theory that there was a true and meaningful difference? E.g., Tre resting change was -0.24C but had a variance of double this (0.48C). This is also observable in the HR section.

Maybe I am misunderstanding some of the analysis that was undertaken?”

**Authors’ response:**

*We appreciate your comment. Our data analysis was strictly ANOVA to T-TEST where appropriate. The data we are using in the present paper is the mean changes. Specifically in the Tre section, we speculate that the variance could be explained by the lesser trained participants experiencing greater magnitude in changes compared to the higher trained individuals (lines 368-373; 386-388).*

**Reviewer’s comment #10:**

“The limitation section is insufficient. I strongly encourage the authors to consider all potential limitations with this present study. For example, the heterogeneity of the type of athletes (endurance, teamsport, strength etc) and indeed the considerable range in performance level (n=5 were DP1 which is ‘untrained’, while n = 5 were ‘trained’!). Acknowledge of these issues does not devalue the importance of the research, rather it allows a reader to gain a better insight into the study outcomes and comparison to other literature.”

**Authors’ response:**

*We appreciate your feedback. We have expanded this section on line 495.*
