# Supplementary material for: Effectiveness of short-term isothermic-heat acclimation (4 days) on physical performance in moderately trained males
Source: PLoS One. 2022 Nov 17;17(11):e0270093. doi: 10.1371/journal.pone.0270093 (PMC9671432; doi:10.1371/journal.pone.0270093)
Supplement: Attachment — Submitted filename: Response to Reviewers.docx [file pone.0270093.s004.docx]

**Reviewer #2:**

**Reviewer’s comment #1:**

“My main issue remains: there is language used within the manuscript, which I do not believe that the results support. For example, Line 289-292. 'post intervention Tre, fc (should be HR), RPE, TC and TS significantly decreased at specific timepoints...' yet based on the results in Lines 252-258, there were only significant effects for time (not day or interaction). Similar data is presented for HR. This is further evidenced in the Figures 4 and 6 which should no pairwise differences. So I question where the sig. differences of Line 289-290 are?”

**Authors’ response:**

*We thank you for your comment. In response to the language used in the manuscript, we have tamed it somewhat and have removed material concerning descriptive statistics from the discussion section – with the exception of showing what was found. We have kept them in the results section so the reference to resting means initially are informed by the results. The significance of Tre and Hr at time points was not meant to be included in our last response. We followed your advice with our ANOVAs and found there was no significance warranting further post-hoc comparisons. We have corrected this immediately.*

**Reviewer’s comment #2:**

“I raised this issue in a previous comment (response #7 and #9) but the author response did not assuage my concerns. I appreciate that the descriptive data were kept, but it should not be discussed as it there were sig.differences between these physiological values across the intervention.”

**Authors’ response:**

*We appreciate your feedback. We have limited the descriptive statistics from the discussion section and rewritten a section of the rectal temperature section while simultaneously taming the language used.*

**Reviewer’s comment #3:**

“Line 257 - remove the word 'in' after the bracket.”

**Authors’ response:**

*We thank you for your feedback. This has been corrected.*

**Reviewer’s comment #4:**

“Line 122 and 289 - fc should be HR”

**Author’ response:**

*We appreciate your thoroughness, and this has been rectified.*

**Reviewer’s comment #5:**

“Line 303 - remove '?'. Also this sentence doesn't read particularly well with the preceding sentence.”

**Authors’ response:**

*We thank you for your comment and have reworked the sentence in question.*

**Reviewer’s comment #6**

“Line 336 - refers to Fig 3 but this appears to be the wrong figure?”

**Authors’ Response:**

*Your comment is appreciated and we have corrected this issue.*

**Reviewer’s comment #7:**

“The abstract mentions results and findings that aren't discussed in the manuscript? E.g., at 70mins, Tre was low pre- to post intervention?? Similarly, the conclusion to the abstract is incorrect based on the present manuscript.”

**Authors’ response:**

*We appreciate your patience with this issue. We have corrected this oversight and reworked the conclusion per you’re your comments.*

**Reviewer’s comment #8:**

“The limitations paragraph is somewhat challenging to understand in the present format; it seems a little disjointed. For example 'we controlled for heat but not exercise' - I assume this refers to the lack of a training control maybe? Needs to be expanded upon. The next sentence also needs clarification. The final sentence doesn't even finish correctly, it just ends partway through a name: University of....? I would suggest that this paragraph be revised to enhance the readability.”

**Authors’ response:**

*We thank you for your comment. We have expanded on the extent of our control. We are not sure why but the latter half of that sentence was hidden but this has been corrected.*
